# Patterns of production of collagen‐rich deposits in peripheral nerves in response to injury: A pilot study in a rabbit model

**DOI:** 10.1002/brb3.659

**Published:** 2017-05-24

**Authors:** Michael Rivlin, Andrew Miller, Jacob Tulipan, Pedro K. Beredjiklian, Mark L. Wang, Jolanta Fertala, Andrzej Steplewski, James Kostas, Andrzej Fertala

**Affiliations:** ^1^ Department of Orthopaedic Surgery Sidney Kimmel Medical College Thomas Jefferson University Philadelphia PA USA; ^2^ Rothman Institute of Orthopaedics Thomas Jefferson University Hospital Philadelphia PA USA

**Keywords:** neural regeneration, neural scarring, neurosurgery, peripheral nervous system, trauma

## Abstract

**Introduction:**

Although collagen‐rich deposits are the main component of neural scars, the patterns of their formation are ill defined. Essential to the biosynthesis of collagen fibrils are enzymes catalyzing posttranslational modifications and chaperones that control the formation of the collagen triple helix. Prolyl‐4‐hydroxylase (P4H) and heat shock protein‐47 (HSP47) play a key role, and their production is upregulated during scar formation in human tissues. Alpha smooth muscle actin (αSMA) is also produced during fibrotic processes in myofibroblasts that participate in fibrotic response. In injured peripheral nerves, however, the distribution of cells that produce these markers is poorly understood.

**Methods:**

The goal of this study was to determine the distribution of the αSMA‐positive, HSP47‐positive, and the P4H‐positive cells to better understand the formation of collagen‐rich fibrotic tissue (FT) in response to peripheral nerve injury. To reach this goal, we employed a rabbit model of crush‐injury and partial‐transection injury of the sciatic nerves.

**Results:**

Our study demonstrated that αSMA is expressed in a relatively small number of cells seen in neural FT. In contrast, cells producing P4H and HSP47 are ubiquitously present in sites of injury of the sciatic nerves.

**Conclusion:**

We contemplate that these proteins may serve as valuable markers that define fibrotic activities in the injured peripheral nerves.

## 1 INTRODUCTION

Peripheral nerve injury (PNI) occurs in an estimated 2%–2.8% of patients with upper and lower extremity trauma, causing a significant burden of disease and disability (Noble, Munro, Prasad, & Midha, 1998). The morbidity associated with PNI is compounded by a high rate of incomplete recovery, with studies reporting good or excellent motor recovery rates as low as 75% for isolated upper extremity nerve lacerations in an ideal setting, and as low as 67% for lower extremity PNI (Kretschmer, Antoniadis, Braun, Rath, & Richter, 2001; Vordemvenne, Langer, Ochman, Raschke, & Schult, 2007). Many of the persistent functional deficits are attributed to neural fibrosis and scarring, which presents a mechanical barrier to peripheral nerve regeneration (Atkins et al., 2006).

The development of perineural scar depends on the type and location of injury. Formation of adhesions to surrounding tissues is termed extraneural fibrosis, whereas fibrotic thickening within the epineurium and endoneurium is termed intraneural fibrosis (Sakurai & Miyasaka, 1986). Scar‐tissue adhesions can affect nerve gliding and mobility, create areas of compression, and impair nerve physiology. In contrast, intraneural scarring results in diversion or blockade of regenerating axons and alters microvasculature of the regenerating nerve (Ngeow, 2010).

Peripheral nerve injury triggers a fibrotic cascade that induces responses of Schwann cells and fibroblastic cells of the epineurium and endoneurium (Burnett & Zager, 2004). During the first 24–96 hr, Schwann cells recruit resident and hematogenous macrophages that also participate in the fibrotic process. These cells are responsible for myelin removal by way of phagocytosis and may also contribute to collagen deposition during fibrosis (Burnett & Zager, 2004; Chen, Piao, & Bonaldo, 2015).

Standardized classifications of PNIs describe the key characteristics of injury sites (Table 1) (Seddon, 1943; Sunderland, 1951). Following a PNI of Seddon‐Sunderland class “2” or higher, Wallerian degeneration occurs within 48–96 hr. For regeneration to be successful, axons must re‐enter the endoneurial tube. This entry, however, is impeded if the ends of the severed nerve produce excessive collagen‐rich scar tissue (Abercrombie & Johnson, 1946).

**Table 1:** Injury classification

Seddon	Sunderland	Pathophysiologic features
Neurapraxia	Type 1	Local myelin damage usually secondary to compression.
Axonotmesis	Type 2	Loss of continuity of axons; endoneurium, perineurium, and epineurium intact.
	Type 3	Loss of continuity of axons and endoneurium; perineurium and epineurium intact.
	Type 4	Loss of continuity of axons, endoneurium, and perineurium; epineurium intact.
Neurotmesis	Type 5	Complete physiologic disruption of entire nerve trunk.

Integral to the biosynthesis of functional collagen molecules are enzymes catalyzing posttranslational modifications of procollagen chains and protein chaperones controlling the formation of the collagen triple helix. Most notably, prolyl‐4‐hydroxylase (P4H), a tetramer consisting of catalytic α and noncatalytic β subunits, catalyzes the hydroxylation of certain proline residues needed for the formation of a stable collagen triple helix. Protein chaperones transiently bind nascent procollagen chains and stabilize them during this formation. In particular, heat‐shock protein 47 (HSP47), a collagen‐specific protein chaperone plays a key role. Studies demonstrated that its production is upregulated during scar formation in a variety of human tissues, including injured joint capsules (Steplewski et al., 2016).

Alpha smooth muscle actin (αSMA), a marker for myofibroblasts, is widely expressed in various tissues during the fibrotic processes (Tomasek, Gabbiani, Hinz, Chaponnier, & Brown, 2002). In uninjured peripheral nerves, αSMA is present in pericytes surrounding blood vessels and within a layer of cells forming the perineurium (Joseph et al., 2004). In injured peripheral nerves, however, the distribution of αSMA‐positive fibroblastic cells is less understood. Similarly, the patterns of expression of HSP47 and αβP4H are not well recognized in damaged peripheral nerves. Thus, the goal of this study was to determine the distributions of the αSMA‐positive, HSP47‐positive, and the αβP4H‐positive cells in response to PNI, in order to better understand the formation of collagen‐rich fibrotic tissue (FT) and to identify targets for potential therapeutic blockade of this process. To achieve this goal, we employed rabbit models of crush‐injury and partial‐transection injury of the sciatic nerves.

## 2 MATERIALS AND METHODS

### 2.1 Nerve injury models

**Figure 1:**
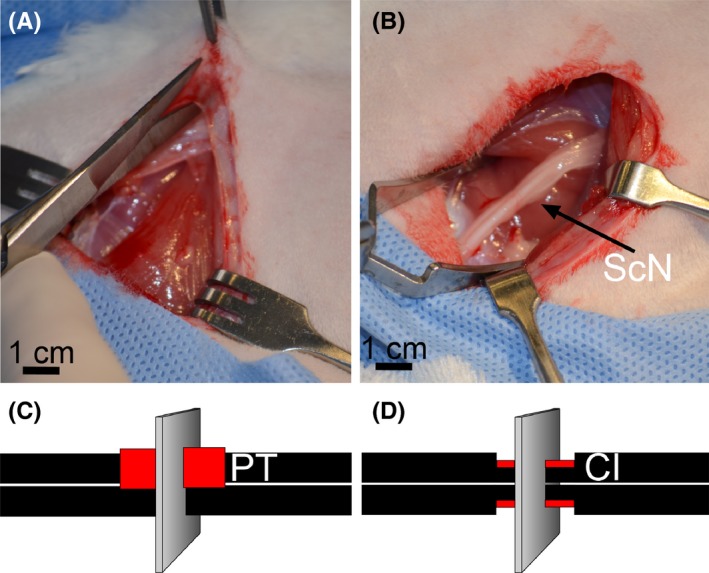
An experimental model of nerve injury. (A,B) Accessing the sciatic nerve of a rabbit. (C) PT nerve injury in which 50% of the thickness of the nerve for a length of 1 cm is removed. (D) CI nerve injury in which a hemostat is placed around the sciatic nerve 1 cm distal to the sciatic notch. The hemostat is then tightened to the first locking flange and held in place for 30 s. In panels C and D, the sites of injury are indicated by red color. Planes corresponding to the orientation of samples collected from the injured nerves are also indicated

All animal studies were approved by the Institutional Animal Care and Use Committee of Thomas Jefferson University. Employing 8‐ to 12‐month‐old White New Zealand female rabbits, we created two injury types to the sciatic nerves: (i) partial‐transection (PT) and (ii) crush‐injury (CI) (Figure 1). In brief, under general anesthesia, a muscle‐sparing approach with minimal bleeding was utilized to access the proximal sciatic nerve of the right leg. In the PT group, a #15 scalpel was used to excise 50% of the diameter of the nerve 1 cm proximal to the sciatic notch. In the CI group, a hemostat was placed around the sciatic nerve 1 cm distal to the sciatic notch. The hemostat (5‐mm width) was then tightened to the first locking flange and held in place for 30 s and repeated distal to cover a 10‐mm length. Before closing the incisions, the injury sites were marked with 8–0 nylon sutures placed in the surrounding tissue to facilitate later identification. Sham surgeries were performed on the left legs by utilizing the same approach to the nerve, and neuroplasty was performed in a similar fashion to the experimental side. The nerve was not manipulated or directly handled. Following surgery, the rabbits were allowed unrestricted cage activity. Animals from the PT and CI groups were then sacrificed at 1 week post‐injury and at 2 weeks post‐surgery. Subsequently, both the injured and uninjured sciatic nerves were harvested for histological analyses.

### 2.2 Processing nerve tissue

The main focus of the microscopic assays was the cross‐sections of the mid regions of the injury sites (Figure 1). We dissected these regions from injured segments defined by nylon sutures placed during surgeries. To stabilize the sciatic nerves, they were embedded in a gelatin‐agarose mixture (Jones & Calabresi, 2007). The embedded samples were fixed in 4% paraformaldehyde and then dehydrated. Next, 3‐mm segments were excised from the center of injury sites and then embedded in paraffin blocks.

### 2.3 Histological assays

To analyze the general morphology of the injury sites, the samples were stained simultaneously with luxol fast blue for myelin and with Sirius red for collagen deposits (Carriel, Garzon, Alaminos, & Campos, 2011). Following this double‐staining, the samples were counterstained with hematoxylin. A microscope (Eclipse E600, Nikon, Inc.) equipped with a digital camera (DS‐Fi1, Nikon, Inc.) was employed to capture the digital images of analyzed samples. In addition, a set of samples stained only with Sirius red was prepared to observe collagen fibrils in polarized light (Steplewski et al., 2016). This was achieved by employing a polarizing microscope (Eclipse LV100POL, Nikon Inc.) equipped with a digital camera (DS‐Fi2, Nikon, Inc.).

### 2.4 Immunohistology

The qualitative and quantitative immunohistological assays were performed to identify cells expressing key proteins associated with collagen production in response to nerve injury. Table 2 lists all antibodies employed in our study and highlights the experimental conditions applied. Following processing for immunofluorescence, all samples were treated with 4′,6‐diamidino‐2‐phenylindole (DAPI) to visualize the nuclei. In all assays, the specimens from injured and healthy nerves were processed simultaneously to ensure identical conditions for immunostaining. A fluorescence microscope (Eclipse E600) equipped with a digital camera (DS‐Qi1Mc, Nikon, Inc.) was employed to visualize and capture images of analyzed regions of injury sites. Corresponding negative controls, in which primary antibodies were omitted, were also prepared for all analyzed samples.

**Table 2:** Primary and secondary antibodies used to detect selected targets

Target	Primary antibody: Host Manufacturer Dilution/incubation	Secondary antibody: Host Manufacturer Dilution/incubation Fluorophore/chromophore
HSP47	Mouse Santa Cruz Biotechnology, Inc. 1:500/4°C/ON	Goat LifeSciences/Thermo Fisher Scientific 1:1,000/RT/1 hr Alexa Fluor 594
αP4H	Goat LifeSpan BioSciences, Inc. 1:200/RT/2 hr	Donkey LifeSciences/Thermo Fisher Scientific 1:1,000/RT/1 hr Alexa Fluor 594
βP4H	Mouse LifeSpan BioSciences, Inc. 1:200/RT/2 hr	Goat LifeSciences/Thermo Fisher Scientific 1:1,000/RT/1 hr Alexa Fluor 594
αSMA	Mouse Abcam 1:200/RT/2 hr	Goat LifeSciences/Thermo Fisher Scientific 1:1,000/RT/1 hr Alexa Fluor 594
S100β	Goat Santa Cruz Biotechnology, Inc. 1:100/4°C/ON	Donkey LifeSciences/Thermo Fisher Scientific 1:1,000/RT/1 hr Alexa Fluor 488
CD68	Mouse Abcam 1:200/4°C/ON	Proprietary 1:500/RT/15 min CSA II Biotin‐Free Tyramide Signal Amplification Kit DAKO 3,3′‐diaminobenzidine (DAB)

We quantified the relative number of cells participating in the production of collagen in response to nerve injury by analyzing the densities of HSP47‐positive and αβP4H‐positive cells present in selected areas of the nerves. To visualize P4H, we targeted both the α and β subunit of this enzyme. Please note that both of these subunits provides equally valid marker for P4H.

Quantification was done with the use the NIS Elements image analysis software (Nikon Inc.). In brief, specific areas of the nerves, e.g., fascicles, epineurium, or extraneural FT, were first selected by outlining regions of interest (ROI). Subsequently, the area occupied by cells positive for a specific marker was determined. The area occupied by cells is a function of their number. Finally, the density of positive cells present in an ROI was expressed as the percent of the total area of the analyzed ROI. A minimum of three sections of the injured and control nerves was used to measure each marker.

### 2.5 Data analysis

Two rabbits per PT and per CI groups were employed in this pilot study. Although randomization should theoretically yield groups that are comparable at baseline, here this might not be the case, as we randomized a relatively small number of animals. To guard against this possibility, we analyzed each outcome in the context of measurements obtained for the injured side versus the measurement obtained for the healthy side, in effect, using each animal as its own control. The Student's *t*‐test was employed to determine the statistical significance of differences between the control group mean and the experimental group mean (GraphPad Prism v. 5.03, GraphPad Software, Inc.).

## 3 RESULTS

### 3.1 Morphology of injury sites

**Figure 2:**
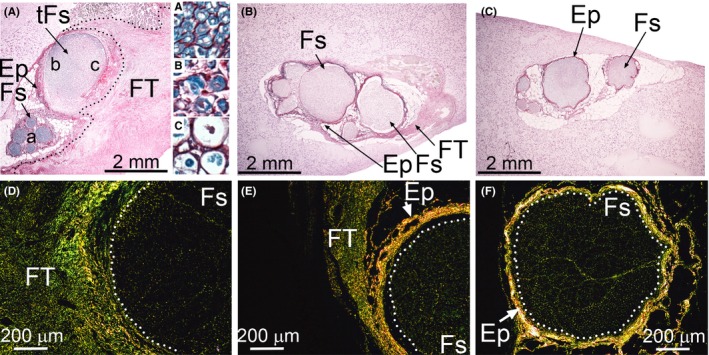
Morphology of the PT and CI nerves. Cross‐sections of the PT (A,D), CI (B,E), and uninjured nerves (C,F) were stained with luxol fast blue for myelin and with Sirius for collagen deposits. While in A, B, and C specimens were observed in regular light, in D, E, and F the presence of Sirius‐stained collagen fibrils was demonstrated with the use of a polarizing microscope. A dotted line in A delineates the FT. Dotted lines in D, E, and F delineate the Fs regions. In panel A, inserts “A”, “B”, and “C” show detailed views of the corresponding a, b, and c regions of depicted fascicles. Symbols: Fs, fascicles; tFs, fascicles located close to a site of injury; Ep, epineurium; FT, fibrotic tissue

Two weeks following PT, significant extraneural FT had formed, and the Sirius red‐positive staining was clearly apparent in the collagen‐rich deposits in this tissue (Figure 2A,D). The epineurium surrounding the fascicles located close to the injury site was dilated compared to those located far from the injury site. In the corresponding CI group, atypical extraneural FT was also observed, but its extent was less prominent than in the PT group (Figure 2B,E). The FT formed around injured nerves was rich in collagen fibrils, which were visible in polarized light (Figure 2D,E).

Samples collected in the PT and CI groups at 1 week post‐injury also contained extraneural FT. This tissue, however, was less developed compared to that of the 2‐week‐post‐surgery counterparts. In contrast, in uninjured control nerves, no FT‐like structures were observed (Figure 2C,F).

In the PT group, the fascicles in the area distal to the transection site appeared morphologically normal (Figure 2A, insert “A”). Myelin appeared intact in some areas of the fascicles adjacent to the injury site (Figure 2A, insert “B”), but other areas adjacent to the injury sites were characterized by extensive myelin degradation demonstrating a gradient of abnormal to normal morphology (Figure 2A, insert “C”).

**Figure 3:**
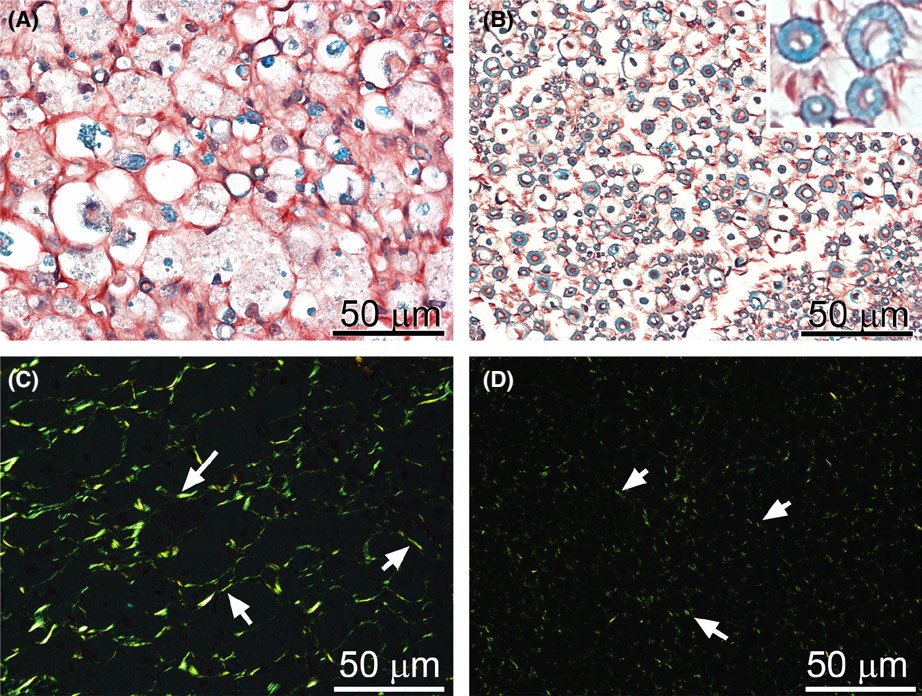
Morphology of the CI nerves stained with luxol fast blue for myelin and with Sirius for collagen deposits. In a crushed nerve (A), degradation of myelin is clearly apparent; in uninjured nerve, abundant myelinated axons are present (B, insert). Observation of Sirius‐stained samples in a polarizing microscope demonstrated an increase in collagen fibrils in the endoneurium of injured nerves (C, arrows). In contrast, in the control, collagen fibrils were sparsely distributed throughout endoneurial space (D, arrows)

In the CI group, areas of myelin degradation were uniformly distributed through the entire fascicular region of the crushed site (Figure 3A). Moreover, a readily visible accumulation of collagen‐rich material was evident within the endoneurium (Figure 3A,C). In contrast, in the sciatic control nerve isolated from the contralateral leg, myelin was intact, and the collagen fibrils seen within endoneurium were less abundant (Figure 3B,D).

### 3.2 Macrophages

**Figure 4:**
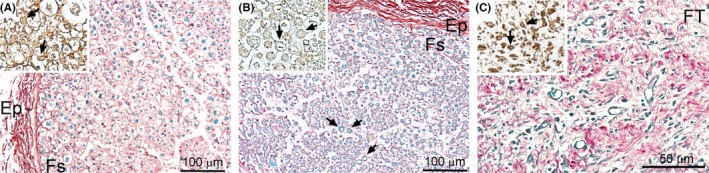
CD68‐positive staining indicating a presence of macrophages in defined regions of the CI and the PT nerves. Macrophages were abundant in crushed nerves in the areas of myelin degradation (A, arrows), whereas in the control no CD68‐positive macrophages were observed around myelinated axons (B, arrows). In the PT injury sites, CD68‐positive macrophages (arrows) were readily observed in perineural fibrotic tissue (C; arrows). While Sirius red‐luxol fast blue‐hematixylin staining depicts the general morphological features of the analyzed regions, the inserts present CD68‐specific staining of macrophages. Symbols: Fs, fascicles; Ep, epineurium; FT, fibrotic tissue

We observed a prominent presence of macrophages in fascicles of the injured nerves isolated from the CI and PT groups. In the nerves from the CI group, the macrophages were distributed throughout the entire crush‐injury site, including the fascicle (Fs) regions. The presence of these cells was closely related with the sites of myelin degeneration within the Fs (Figure 4A). In corresponding sites of uninjured nerves, macrophage‐specific staining was not apparent (Figure 4B). In nerves from the PT model, the macrophages were readily visible in the fibrotic (FT) region (Figure 4C).

### 3.3 αSMA, HSP47*,* αβP4H

**Figure 5:**
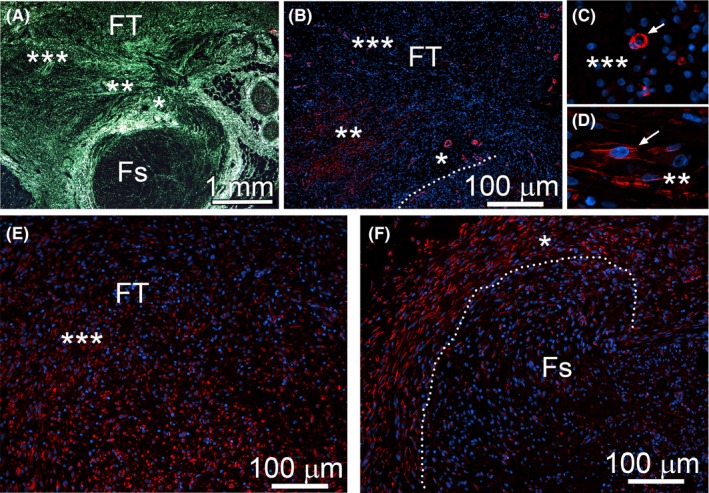
Analysis of the distribution of αSMA‐positive cells (B–D) and HSP47‐positive cells (E,F) in the PT nerves. In panels B, C, D, E, and F asterisks indicate corresponding regions depicted at lower magnification in panel A. A, Sirius red staining to visualize collagen‐rich region of the PT nerve. B, αSMA‐positive cells seen in regions defined in A by asterisks. (C,D) Magnification of selected regions; in C αSMA‐positive pericytes are indicated (arrow), and, in D fibroblastic αSMA‐positive cells are indicated (arrow). Dotted lines delineate a fascicle. Symbols: FT, fibrotic tissue; Fs, fascicle

We analyzed αSMA to identify myofibroblastic cells present in nerve injury sites. Employing Sirius red staining to visualize collagen‐rich areas, close microscopic examination of corresponding regions (Figure 5, asterisks) in the PT nerves revealed groups of αSMA‐positive cells in the FT that localized mainly within areas close to the fascicles adjacent to the site of injury (Figure 5B,D). αSMA‐positive cells were not readily apparent in remaining areas of the FT, except blood vessels (Figure 5B,C). In contrast, HSP47‐positive cells (Figure 5E,F) were present throughout the entire FT region. The pattern of distribution of the αβP4H‐positive cells (not shown) was essentially identical to that seen fror HSP47 (Figure 5E,F).

**Figure 6:**
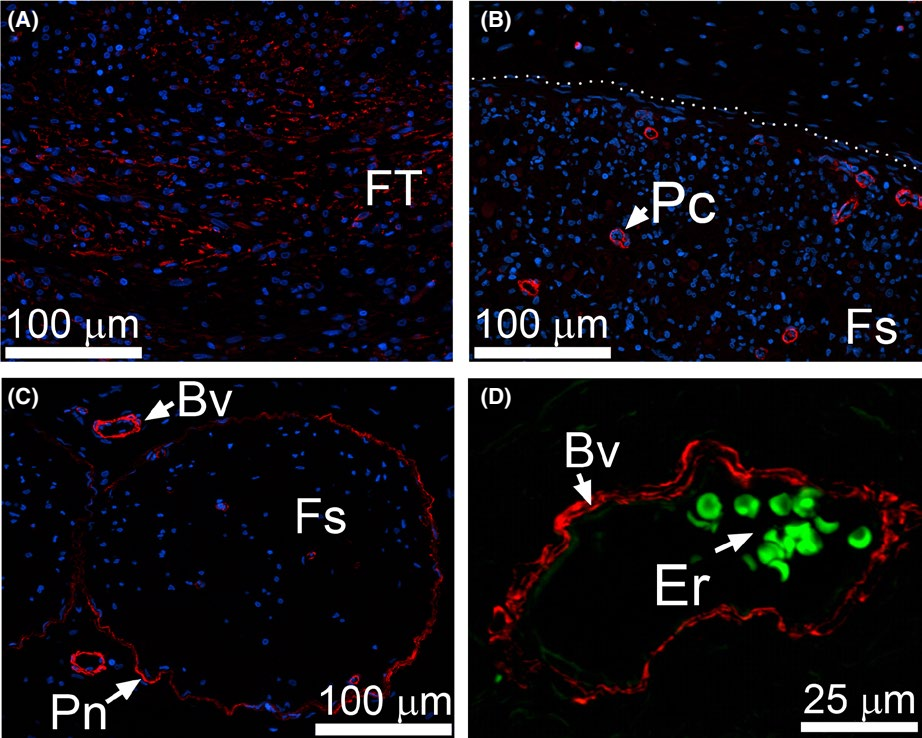
αSMA immunostaining of the CI nerves. (A) αSMA‐positive cells are evident in perineural FT. (B) Within fascicles (delineated with a dotted line) αSMA‐positive staining is only seen in pericytes (Pc). (C) In uninjured control αSMA‐positive staining is also observed around endoneurial blood vessels (Bv) and within perineurium (Pn). (D) A high magnification of a blood vessel identified by the presence of erythrocytes (Er) seen due to green autofluorescence

Similarly, in the CI nerves, we observed αSMA‐positive cells only in the extraneural FT formed due to injury (Figure 6A) and around blood vessels identified by the presence of erythrocytes whose green autofluorescence is caused by peroxidation (Figure 6D) (Khandelwal & Saxena, 2007). We noted αSMA‐positive cells in the perineurium of uninjured control nerves (Figure 6C), but not in the corresponding region of CI nerves (Figure 6B).

**Figure 7:**
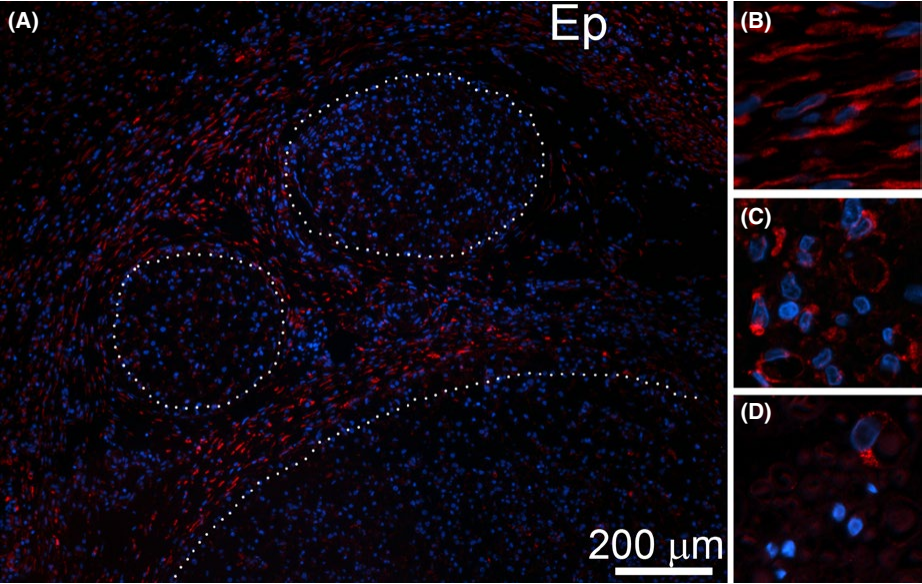
HSP47‐positive cells are evident within the CI nerves. (A) A representative region depicting fascicles (delineated with dotted lines) and epineurium (Ep). (B) A magnified view at HSP47‐positive cells present in epineurium of a CI site. (C) A magnified view at HSP47‐positive cells present within endoneurium of a CI site. (D) A magnified view at HSP47‐positive cells present within endoneurium of a control uninjured nerve

**Figure 8:**
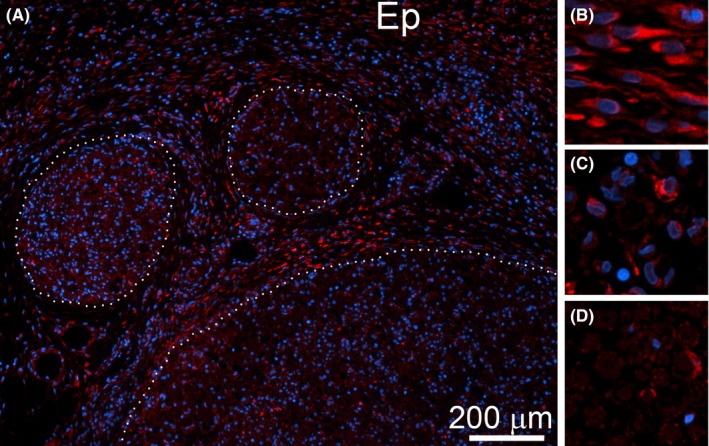
αP4H‐positive cells are evident within CI nerves. (A) A representative region depicting fascicles (delineated with dotted lines) and epineurium (Ep). (B) A magnified view at αP4H‐positive cells present in epineurium of a CI site. (C) A magnified view at αP4H‐positive cells present within endoneurium of a CI site. (D) A magnified view at αP4H‐positive cells present within endoneurium of a control nerve

Cells seen in the CI nerves were HSP47‐positive (Figure 7) and αβP4H‐positive (Figure 8, note that only the αP4H‐specific staining is presented). In addition to epineurium, HSP47‐positive (Figure 7) and αβP4H‐positive cells (Figure 8, note that only aP4H‐specific staining is presented) were also present within fascicles of the CI nerves. Although present within fascicles of control uninjured nerves, HSP47‐positive (Figure 7D) and αβP4H‐positive cells (Figure 8D, note that only αP4H‐specific staining is presented) were less abundant than in the injured counterparts.

**Figure 9:**
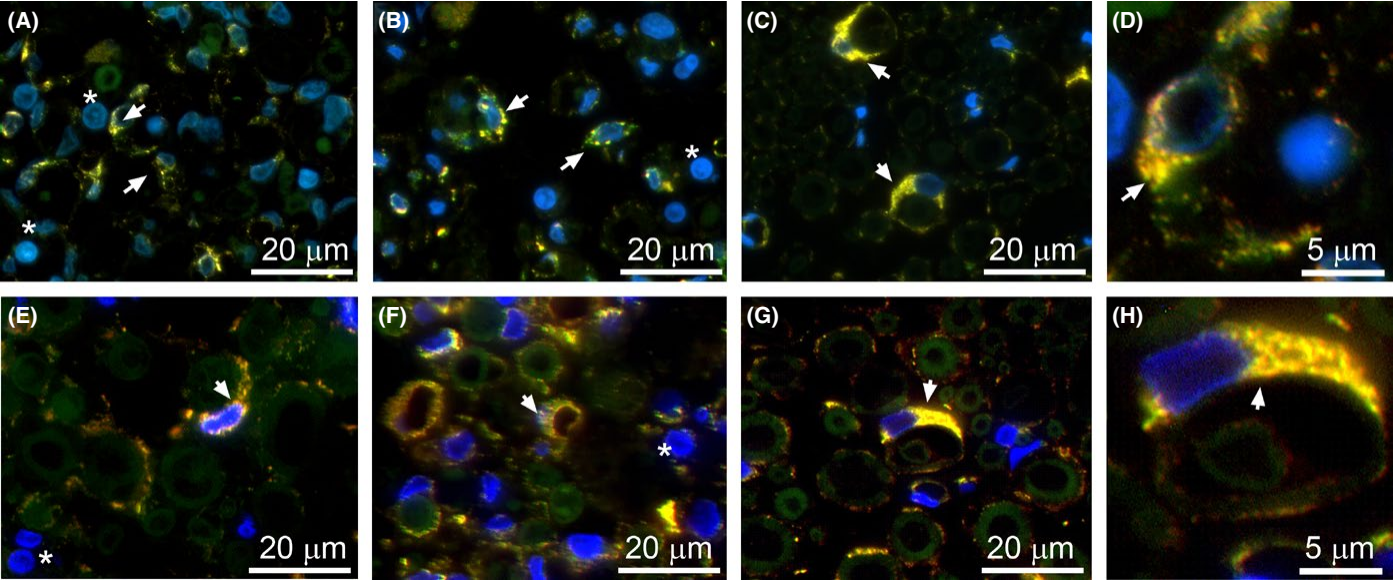
Double staining to co‐localize HSP47/S100β‐positive (A–D) and βP4H/S100β‐positive (E–H) cells. Fascicles of a PT nerve (A,E), CI nerve (B,F), and control (C,G) are depicted. High‐magnification panels showing details of double staining are also included (D,H). Yellow color indicates co‐localization of analyzed markers (arrows). Note abundant macrophages in panels A, B, E, and F (asterisks)

Employing double‐immunostaining with anti‐HSP47/anti‐S100βantibodies or anti‐βP4H/anti‐S100βantibodies, we determined that, within fascicles seen in the PT and CI nerves, the HSP47‐positive (Figure 9A,B) and βP4H‐positive cells (Figure 9E,F) were Schwann cells. Due to damage of the axons, the morphology of these cells changed (Figure 9A,B,E,F). In contrast, in non‐injured nerves, the Schwann cells maintained their association with the axons (Figure 9C,G).

### 3.4 Time‐dependent changes

We analyzed the distribution patterns of HSP47‐positive and αβP4H‐positive cells in the PT and CI nerves 1 week (1‐W) and 2 weeks (2‐W) after injury. Within PT nerves, the following areas were defined: (i) fascicle; Fs, (ii) altered fascicle area close to transection site; tFs, (iii) epineurium; Ep, and (iv) extraneural fibrotic tissue; FT. While the tFs region was clearly apparent in the 2‐W group (Figure 2), its presence in the 1‐W group was not readily visible. Within the CI nerves, the following areas were defined: (i) fascicle (Fs); (ii) epineurium (Ep); and (iii) extraneural fibrotic tissue (FT).

**Figure 10:**
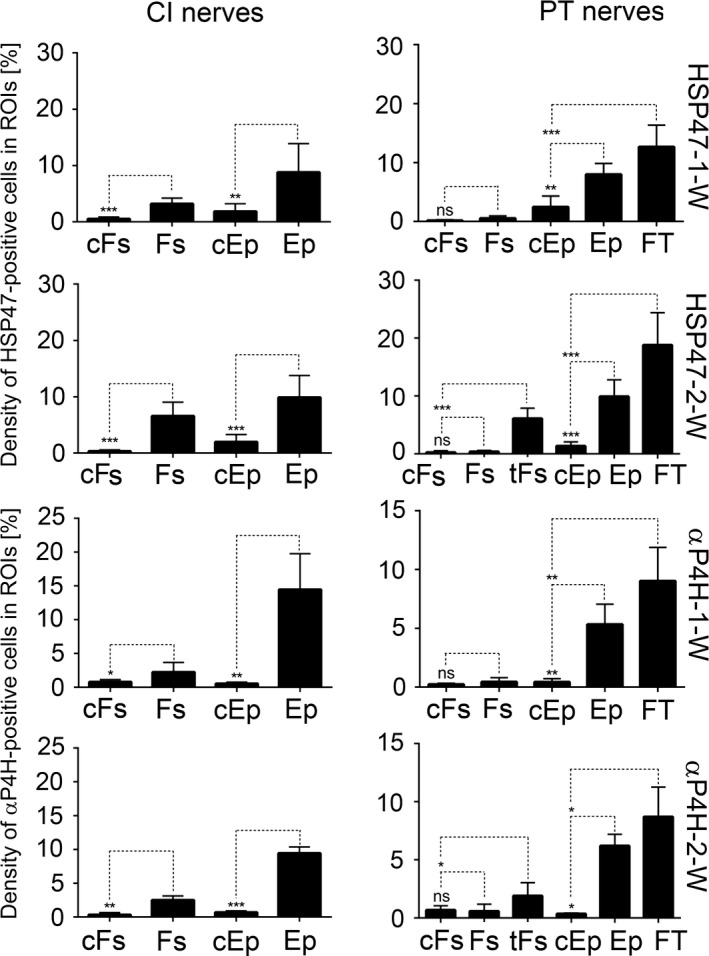
A graphic representation of a relative cell number expressed as percent of area occupied by them within defined ROIs. cFs, fascicles from control uninjured nerves; tFs, altered fascicle area located close to transection site; Fs, fascicles from injured nerves; cEp, epineurium from control uninjured nerves; Ep, epineurium from injured nerves; FT, fibrotic tissue. *p<0.05, **p<0.01, ***p<0.001

Quantification of the density of HSP47‐positive and αβP4H‐positive cells present in the analyzed regions of injured nerves revealed remarkable changes compared to the corresponding areas of the non‐injured controls. Since the changes for the αP4H and the βP4H subunits were identical, we only present results for the α subunit (Figure 10). Specifically, in the CI nerves, we observed a statistically significant increase in the density of cells expressing the analyzed markers in the Fs and the Ep regions. In the PT group, we observed no changes between the Fs regions of uninjured and injured groups. In the tFs region, however, the increase in the HSP47‐positive and αβP4H‐positive cells was statistically significant compared to the Fs control. Similarly, a statistically significant increase was observed in the FT area compared to the Ep area of control. Figure 10 depicts a graphical representation of the described changes (note that only αP4H‐specific data are presented).

## 4 DISCUSSION

Although collagen fibrils are crucial elements of a proper architecture of peripheral nerves, upregulation of their production due to injury may alter nerve regeneration and impede its function (Atkins et al., 2006). Collagen fibrils are products of cells that biosynthesize collagen molecules in a complex process that includes intracellular and extracellular steps. In addition to the Schwann cells, peripheral nerves include epineurial fibroblasts, perineurial cells, pericytes, and endoneurial fibroblasts. Moreover, mast cells and resident macrophages are also present (Hall, 2005). Researchers suggested that similar to the Schwann cells, endoneurial fibroblasts may also originate from neural crest stem cells (Jessen et al., 1994; Joseph et al., 2004). Unlike Schwann cells, endoneurial fibroblasts do not associate with axons and fail to express S100β in vivo. Moreover, in contrast to pericytes, these cells do not express αSMA (Joseph et al., 2004).

Although Schwann cells and fibroblastic cells of peripheral nerves produce collagenous proteins in physiological conditions, the specific contribution of these cells in production of collagen‐rich deposits formed in response to nerve injury is not clear (Chernousov & Carey, 2000). Since the production of functional collagens depends on a complex machinery of the auxiliary proteins that must modify nascent collagen chains before they form functional collagen molecules and then complex fibrils, we analyzed the patterns of expression of HSP47 and αβP4H. We contrasted the patterns of expression of HSP47 and P4H with those for αSMA to evaluate the utility of these markers to define the active sites of collagen production and neural scar formation.

In the PT model, the collagen‐rich deposits were largely produced within the extraneural FT. We observed that only some of the cells present in the FT were positive for αSMA. As αSMA is expressed by myofibroblastic cells that contribute to fibrillar collagen production in a number of FTs, it is quite remarkable that only a relatively small number of cells present in the extraneural FT were αSMA‐positive. Similarly, epineurial fibroblasts located close to the fascicular region were essentially αSMA‐negative. As fibroblasts differentiate into myofibroblasts in response to increasing tension of surrounding extracellular matrix, it is likely that in our models mechanical cues needed for this differentiation exist only in selected regions of the neural FT (Tomasek et al., 2002).

In contrast to αSMA, the cells expressing HSP47 and αβP4H were abundant throughout entire FT, including a region bordering the adjacent fascicles. This may indicate a non‐myofibroblastic subset of collagen‐producing cells actively participating in neural fibrosis. Moreover, HSP47 and αβP4H were also expressed in cells seen within the tFs regions located within the fascicles adjacent to the FT site. The area where these cells were present overlapped with infiltrating macrophages and coincided with regions of myelin degeneration. Our studies, however, did not show any apparent expression of HSP47 and αβP4H in the macrophages participating in nerve repair. Although macrophages may produce collagen VI in response to nerve injury, this production depends on their activation status and differentiation stage (Chen, Cescon, Megighian, & Bonaldo, 2014; Schnoor et al., 2008; Stratton & Shah, 2016). Thus, the absence of HSP47 and αβP4H in these cells indicates that the expression of collagen VI in macrophages is, most likely, not active at the 1‐week and 2‐week post‐injury time points selected in this study.

In the FT and epineurial areas of the nerves from the CI group, the distribution pattern of the αSMA‐positive cells was essentially similar to that in the PT group. Specifically, these cells were mainly present in the extraneural FT region. In contrast, the HSP47‐positive and the αβP4H‐positive cells were readily visible in the Ep regions and the Fs regions.

In the tFs regions of the PT and the Fs regions of the CI nerves, we showed that the HSP47‐positive and the αβP4H‐positive cells are the Schwann cells. In the PT nerves, the HSP47/S100β‐positive cells were mainly present in the tFs areas, while in the CI nerves, double‐stained cells were seen through the entire Fs areas. This may indicate that Schwann cells not only maintain the homeostasis of collagenous matrices in physiological conditions, but they also accelerate collagen production in response to the nerve injury, thereby contributing to neural fibrosis.

Although αSMA plays a role in the mechanism generating neuroma‐associated pain, our study indicates that this protein is not a reliable marker of areas of neural fibrosis (Weng et al., 2016). In contrast, the accelerated expression of HSP47 and αβP4H in fibroblastic cells and Schwann cells clearly suggests their pro‐fibrotic activity in response to nerve injury and defines areas of collagen deposition. Because of the ubiquitous presence of HSP47 during fibrotic processes, assays of its expression at the mRNA and protein levels may offer a powerful diagnostic tool to monitor the progression, the extent, and a potential regression of neural fibrosis in response to anti‐fibrotic treatments (Taguchi & Razzaque, 2007).

There are a few limitations of this study that have to be addressed in the future to fully assess the utility of auxiliary proteins associated with collagen production to serve as markers of neural fibrosis. First, larger groups of animals will have to be employed to evaluate extensively the significance of measured parameters as indicators of neural fibrosis. Second, yet another limitation of our study is that our assays of the expression patterns of αSMA, HSP47 and αβP4H were done at relatively late post‐injury stages. Earlier studies showed that increased proliferative and metabolic activities of cells that participate in regeneration of peripheral nerves start as early as day one post‐injury and may continue weeks after the initial trauma (Jurecka, Ammerer, & Lassmann, 1975). Thus, to fully determine the utility of HSP47 and αβP4H as indicators of neural fibrosis, further studies are warranted to study their expression patterns during early post‐injury stages.

## CONFLICT OF INTEREST

None declared.
